# Carcinomembrane-Camouflaged Perfluorochemical Dual-Layer Nanopolymersomes Bearing Indocyanine Green and Camptothecin Effectuate Targeting Photochemotherapy of Cancer

**DOI:** 10.1021/acsbiomaterials.4c01150

**Published:** 2024-09-12

**Authors:** Yu-Hsiang Lee, Cai-Sin Chen

**Affiliations:** † Department of Biomedical Sciences and Engineering, National Central University, Taoyuan City 32001, Taiwan R.O.C; ‡ Department of Chemical and Materials Engineering, National Central University, Taoyuan City 32001, Taiwan R.O.C

**Keywords:** anticancer, photochemotherapy, cancer cell membrane, perfluorocarbon nanopolymersomes, NIR irradiation, homologous targeting

## Abstract

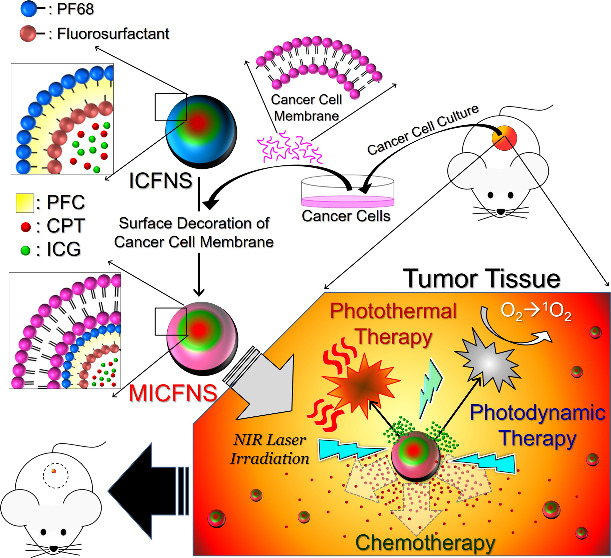

Photochemotherapy has been recognized as a promising combinational modality for cancer treatment. However, difficulties such as off-target drug delivery, systemic toxicity, and the hypoxic nature of the tumor microenvironment remain hindrances to its application. To overcome these challenges, cancer cell membrane camouflaged perfluorooctyl bromide (PFOB) dual-layer nanopolymersomes bearing indocyanine green (ICG) and camptothecin (CPT), named MICFNS, were developed in this study, and melanoma was exploited as the model for MICFNS manufacture and therapeutic application. Our data showed that MICFNS were able to stabilize both ICG and CPT in the nanocarriers and can be quickly internalized by B16F10 cells due to melanoma membrane-mediated homology. Upon NIR irradiation, MICFNS can trigger hyperthermia and offer enhanced singlet oxygen production due to the incorporation of PFOB. With ≥10/2.5 μM ICG/CPT, MICFNS + NIR can provide comparable *in vitro* cancericidal effects to those caused by using an 8-fold higher dose of encapsulated CPT alone. Through the animal study, we further demonstrated that MICFNS can be quickly brought to tumors and have a longer retention time than those of free agents *in vivo*. Moreover, the MICFNS with 40/10 μM ICG/CPT in combination with 30 s NIR irradiation can successfully inhibit tumor growth without systemic toxicity in mice within the 14 day treatment. We speculate that such an antitumoral effect was achieved by phototherapy followed by chemotherapy, a two-stage tumoricidal process performed by MICFNS. Taken together, we anticipate that MICFNS, a photochemotherapeutic nanoplatform, has high potential for use in clinical anticancer treatment.

## 1 Introduction

According to the statistics of the Global Cancer Observatory (GCO) (https://gco.iarc.who.int), cancer remains the leading cause of death in the world, and the burden of cancer incidence and mortality is rapidly rising due to growing and aging global populations, as well as increase of risk factors such as pollution, obesity, and dietary patterns over the last decades. Specifically, there were approximately 20 million new cases of cancer and 10 million deaths from cancer in the year 2022, suggesting that nearly one in five people develop cancer in their lifetime, whereas about one in nine men and one in 12 women die from it.^1^ These circumstances denote that development of an effective strategy for cancer treatment is still highly demanded nowadays.

So far, chemotherapy remains the mainstay of treatment for the majority of solid and hematological malignancies. However, it is known that the hypoxic nature of the tumor microenvironment (TME) can quickly trigger drug resistance of cancer cells and lead to treatment failure,^2^ while increase of drug dosage may induce serious side effects in patients such as hair loss, cardiotoxicity, bone marrow suppression, and/or liver dysfunction.^3^ To overcome these issues, combination of chemotherapy and other modalities is often considered a promising approach because the joint regimen may provide other anticancer mechanisms and/or compensate the cancericidal effects, whereby the dose of the drug employed can be reduced without compromising the overall anticancer efficacy.

Phototherapy, including photothermal therapy (PTT) and photodynamic therapy (PDT), has long been recognized as a feasible adjuvant for chemotherapy due to its unique characteristics such as being noninvasive, having a controlled light delivery process, and producing minor side effects.^4,5^ In general, PTT is accomplished by ablating cancer cells with >48 °C (hyperthermia),^6,7^ while PDT is carried out by impairing cell metabolism, tumoral vasculature, and/or DNA replication through the produced reactive oxygen species (ROS).^8−10^ Nonetheless, the clinical applicability of phototherapy is still hindered by several limitations. For instance, thermal ablation during PTT may damage the surrounding normal tissues and induce serious post-treatment inflammation due to massive heat diffusion and/or release of tumor debris/contents.^11,12^ Furthermore, the hypoxic TME is detrimental to ROS generation and thus restricts PDT effects.^13,14^ Moreover, it is critical to transport both photosensitizers and chemotherapy drugs with appropriate dosages together to tumors to ensure effective photochemotherapy.

Co-administration of multiple agents through nanoencapsulation technology may offer a feasible means to overcome the aforementioned challenges because appropriate nanocarriers can improve the security, stability, and bioavailability of the payloads.^15,16^ However, the circulation time of nanocarriers is highly dependent on their surface properties such as hydrophilicity, zeta potential, and biosimilarity.^17−19^ Among various surface modification methodologies, the biomimetic approach of coating the nanocarrier surface with a cancer cell membrane has been demonstrated as a feasible method to enhance the bioavailability of nanocarriers since the capabilities of cancer cells regarding immune escape and homologous binding are all highly associated with their membrane proteins as reported previously.^20^ Indeed, quite a few studies have shown that decorated cancer cell membranes retain the antigenic diversity of the source cells and thus endow the treated nanocarrier with homology and prolonged retention time *in vivo.*(21−23) Moreover, such a top-down approach dramatically simplifies the procedures of biomimetic modification of nanocarriers.^24,25^

In this study, we sought to develop a type of cancer cell membrane camouflaged perfluorocarbon (PFC) nanopolymersomes carrying indocyanine green (ICG) and camptothecin (CPT), named MICFNS, and explored their potential for photochemotherapy of tumors using skin cancer as the disease model. Near-infrared (NIR) light was employed as the light source because it can provide enhanced penetration depth in tissue compared to visible light.^26,27^ ICG is a USFDA-approved NIR fluorophore which can absorb and fluoresce in the 650–850 nm range and has been widely used in both medical diagnosis^28−30^ and anticancer phototherapy.^31−33^ PFC, a fluorine-substituted derivative of hydrocarbons, was exploited as an oxygen reservoir in MICFNS since it has exceptional oxygen solubility (>30 mmol/L at 25 °C and 1 atm) compared to water (<3 mmol/L at 25 °C and 1 atm).^34^ Taken together, the developed MICFNS are anticipated to be multifunctional nanoagents that can (1) stabilize the entrapped molecules from degradation caused by thermal stimulation, (2) simultaneously deliver both ICG and CPT to tumors with homologous targetability, (3) enhance PDT efficacy due to oxygenation of MICFNS by PFC, and (4) offer remarkable anticancer effectiveness with reduced systemic inflammation or chemotoxicity for anticancer applications. In this work, the fabrication of MICFNS is first introduced, followed by studies of their characteristics, functionalities, and anticancer effects *in vitro* and *in vivo*.

## 2 Materials and Methods

### 2.1 Cell Culture

Murine melanoma B16F10 cells (ATCC, Manassas, VA, USA) were cultured using Dulbecco’s modified eagle medium (DMEM) supplemented with 10% fetal bovine serum (FBS), 1.5 g/L sodium bicarbonate, and 100 U/mL penicillin/streptomycin at 37 °C with 5% CO_2_. Murine hepatocyte FL83B cells (ATCC) were cultured using 90% Ham’s F12K medium supplemented with 10% FBS, 1.5 g/L sodium bicarbonate, 2 mM l-glutamine, and 100 U/mL penicillin/streptomycin at 37 °C with 5% CO_2_.

### 2.2 Preparation of Cancer Cell Membranes

The B16F10 cell membranes (BCMs) was harvested using the Plasma Membrane Protein Extraction Kit (Abcam, Cambridge, UK) according to the manufacturer’s instructions. Briefly, the cells dispersed in a homogenizing buffer with a protease inhibitor cocktail were sonicated at an intensity of 55 W for 15 min under an ice bath, and then the homogenized mixture was centrifuged at 700 × *g* at 4 °C for 10 min. Afterward, the supernatant was collected and centrifuged at 10,000 × *g* at 4 °C for 30 min by which the BCMs can be obtained in the precipitates. The BCMs were resuspended in PBS and subjected to the BCA assay for measurement of protein concentration immediately after collection.

### 2.3 Fabrication and Characterization of MICFNS

The ICG and CPT coloaded PFC nanopolymersomes (ICFNS) were first synthesized through a dual emulsification protocol reported previously.^31^ Briefly, 1 mL of methanol (50 vol %) containing ICG (0.1 wt %, Sigma-Aldrich, St. Louis, MO, USA) and CPT (0.02 wt %, Sigma-Aldrich) was added to 2 mL of perfluorooctyl bromide (PFOB, Sigma-Aldrich) containing 2 wt % fluorosurfactant (Capstone FS-3100, Chemours, Wilmington, DE, USA), and the mixture was homogenized for 6 min in an ice bath. The obtained water-in-PFC emulsions were then added to 12 mL of Pluronic F68 copolymer (PF68, Sigma-Aldrich) solution (5 wt %) and processed with sonication again for 10 min to form ICFNS. After being washed twice with deionized (DI) water, the ICFNS were subjected to surface decoration with BCMs immediately or stored at 4 °C until use.

**Figure 1:**
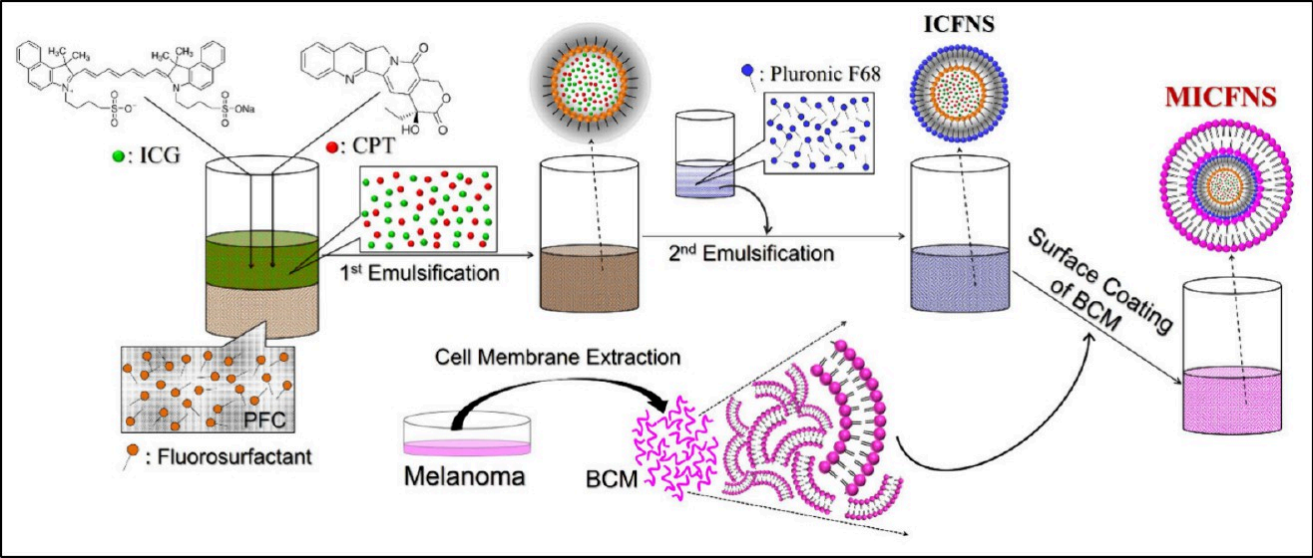
Schematic diagram of the MICFNS fabrication.

The MICFNS were prepared using an extruding approach reported previously.^21^ In brief, the BCMs were first extruded through polycarbonate porous membranes (400 nm, Avanti Polar Lipids, Alabaster, AL, USA) seven times and then immediately added to ICFNS with 1:50 of the weight ratio (w/w; BCM/ICFNS). The mixture was extruded again through polycarbonate porous membranes (400 nm) nine times, whereby the MICFNS were formed thereafter. After being washed twice with DI water, the MICFNS were resuspended in PBS and stored at 4 °C. The fabrication of MICFNS is schematically illustrated in Figure 1.

The morphologies of ICFNS and MICFNS were detected using scanning electron microscopy (SEM) and transmission electron microscopy (TEM) with negative staining by phosphotungstic acid (PTA; 2%). Both the zeta potential and size distribution of the extruded BCMs, ICFNS, and MICFNS were measured by dynamic light scattering (DLS). The loading and encapsulation ratios of ICG and CPT in the MICFNS were evaluated using the spectrophotometric methods reported previously.^31^ Stability of MICFNS under different temperatures was examined by monitoring the size and density of the nanopolymersomes under incubation at 4 or 37 °C for 7 days. For each temperature setting, the mean size of the MICFNS was detected by DLS at day 0, 1, 3, 5, and 7, while their particle number (*Q*) was calculated using the equation
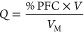
1where %PFC represents the PFOB volume percentage in the MICFNS sample which can be obtained by gravimetric measurement and regression analysis reported previously.^35^*V* is the total volume of the MICFNS sample, while *V*_M_ is the volume of a single ideal spherical MICFNS. The integrity of the BCM on the MICFNS surface was verified by detecting the melanoma membrane markers N-cadherin and CD47 through Western blot, where the BCM of the MICFNS (if available) was collected by a RIPA-mediated approach reported previously.^36^

### 2.4 Assessment of Cellular Uptake Efficiency of MICFNS

B16F10 and FL83B cells were separately treated with ICFNS or MICFNS containing 20/5 μM ICG/CPT at 37 °C for 4 h. Afterward, all cells were washed twice with PBS and immediately subjected to spectrofluorometry set as 750 nm excitation wavelength and 838 nm emission wavelength for measurement of intracellular ICG-derived fluorescence. The expression level of fluorescence was quantitatively represented by relative fluorescence units (RFUs) in this study.

### 2.5 Assessment of Thermal Stability and Drug Release Kinetics of MICFNS

The ICFNS and MICFNS with 20/5 μM ICG/CPT were placed at 4 or 37 °C. After maintenance for 3, 6, 12, 24, and 48 h, the ICFNS and MICFNS, as well as their supernatants, were separately collected through centrifugation at 6,000 × *g* at 4 °C for 30 min. The collected nanopolymersomes and supernatants were separately detected with spectrophotometry at λ = 780 and 370 nm to analyze the amount of ICG remaining in the nanocarriers and the quantity of CPT released to the medium, respectively.

To assess how NIR irradiation influences the effect of drug release, MICFNS with 40/10 μM of ICG/CPT were separately treated with NIR for 1, 2, 3, 4, and 5 min, and then the quantity of CPT in each supernatant was evaluated by spectrophotometry described above. The temperature of the nanopolymersome system under NIR exposure was simultaneously measured every 60 s for 5 min. NIR irradiation was performed using an 808 nm optical laser with an output intensity of 6 W/cm^2^ throughout the study.

### 2.6 Measurements of Hyperthermia Effect and Singlet Oxygen Production of MICFNS

200 μL of MICFNS containing 2.5, 5, 10, 20, 40, and 80 μM ICG in 96-well culture plates were separately irradiated with NIR (808 nm, 6 W/cm^2^), and the temperature of each group was detected every 30 s for 5 min using an infrared thermometer.

The production of singlet oxygen generated from the MICFNS under NIR exposure (808 nm, 6 W/cm^2^) was evaluated using the singlet oxygen sensor green (SOSG) kit (Life Technologies, Carlsbad, CA, USA) according to the manufacturer’s instructions. The doses of ICG in the MICFNS were set as 0 (medium only), 2.5, 5, 10, 20, 40, and 80 μM. The quantity of singlet oxygen in each group was assessed based on the SOSG-induced fluorescence detected every 60 s for 5 min.

### 2.7 Evaluation of Cytotoxicity of MICFNS *iIn Vitro*

B16F10 cells (10^5^ cells/well) in 96-well culture plates were separately treated with NIR, free CPT, free ICG + NIR, MICFNS, and MICFNS + NIR. The concentrations of free ICG and CPT were identical to the doses provided by the MICFNS, and those were set as [ICG]/[CPT] = 0/0, 2.5/0.625, 5/1.25, 10/2.5, 20/5, 40/10, and 80/20 μM. NIR irradiation (808 nm, 6 W/cm^2^) was conducted for 5 min in each well. Cell viabilities were examined using both MTT and calcein-AM/propidium iodide (PI) staining assays 24 h after treatment. The temperature and production of singlet oxygen generated from the ICG and MICFNS used for the cytotoxicity assay were measured using an infrared thermometer and SOSG kit described above after 5 min of NIR irradiation.

### 2.8 Animal Model

C57BL/6 mice (6–7 weeks; 30 ± 5 g) purchased from BioLASCO (Taipei, Taiwan ROC) were used to establish the animal model. Tumor implantation was performed by injecting 1 × 10^7^ B16F10 cells into the flank region of each mouse. The size of the tumor (*V*) was measured every 48 h by *V* = (*L* × *W*^2^)/2 where *L* and *W* represent the tumor length in major and minor axis, respectively. The tumor-bearing mice were ready for experiments when the tumor size reached 80–100 mm^3^. All the animal protocols including daily care and experimental methods followed the guidelines approved by the Institutional Animal Care and Use Committee at the National Atomic Research Institute (Taoyuan, Taiwan ROC, Approval number: 112004).

### 2.9 Hemolytic Analysis *Ex Vivo*

Fresh mouse blood was first collected in an anticoagulant tube containing heparin. Red blood cells were then isolated from the whole blood by centrifugation at 2,500 × *g* in ambient temperature for 10 min, followed by washing twice with PBS. Both ICFNS and MICFNS that each contained 5/1.25 or 40/10 μM [ICG]/[CPT] were incubated with the harvested red blood cells in suspension at 37 °C for 2 h. The concentration of the erythrocytes was set as 5% (v/v) in all groups. Tween X-100 and PBS were employed as the positive and negative controls, respectively. The supernatant of each group after 2 h incubation was collected by centrifugation set at 2,500 × *g* in ambient temperature for 10 min and immediately subjected to detection of hemoglobin concentration using a spectrophotometer (SynergyTM HT, BioTek, Winooski, VT, USA) at λ = 540 nm. The hemolysis ratio of each group was calculated by comparing the absorbance value gained from the positive control.

### 2.10 *In Vivo* Imaging

B16F10 tumor-bearing mice were intravenously injected with free ICG, ICFNS, or MICFNS from the tail (*n* = 3 for each) or intratumorally injected with free ICG or MICFNS (*n* = 3 for each) with ICG concentrations in all the ICG-related agents set as 80 μM for each assay. The levels of ICG-derived fluorescence in the intravenously injected mice were detected after 1, 4, 16, and 24 h, while those in the intratumorally injected mice were measured at day 1, 3, 5, and 7 after injection using the IVIS imaging system (In-Vivo Xtreme II, Bruker, Billerica, MA, USA). Moreover, all of the tumors and five organs (kidney, lung, spleen, liver, and heart) of the latter group were collected after the mice were sacrificed on the seventh day and subjected to fluorescence analyses by IVIS immediately.

### 2.11 Assessment of the Antitumor Effect of MICFNS *In Vivo*

B16F10 tumor-bearing mice were separately treated with PBS, free CPT, free ICG + NIR, MICFNS, and MICFNS + NIR (*n* = 3 for each) and NIR irradiation was applied using an 808 nm laser with an intensity of 6 W/cm^2^ for 30 s. The concentrations of free ICG and CPT were set to be the same as those provided by the MICFNS and were 40 and 10 μM, respectively. Each kind of agent was applied to tumors (100 μL/tumor) intratumorally every 48 h for 14 days. The body weight, appearance, and tumor size of each experimental mouse were detected every 48 h before the next treatment. The tumors and five major organs (lung, spleen, kidney, liver, and heart) of all of the mice were extracted upon sacrifice and subjected to histological and systemic toxicity tests immediately.

### 2.12 Histological Study

Preparation of tissue specimens for histological analysis followed the regular preparation procedures described elsewhere.^37^ The tissues of five organs were subjected to hematoxylin and eosin (H&E) staining, while the tumors were processed with both H&E staining and caspase-3 and Ki-67 immunohistochemical (IHC) assays. All the stained tissues were photographed and observed using Motic DSA software (Motic, Kowloon, Hong Kong). Furthermore, the percentage of the area with caspase-3 or Ki-67 expression was quantitatively analyzed using ImageJ software.

### 2.13 Assessment of Systemic Toxicity of MICFNS

The numbers of platelets (PLT), red blood cells (RBC), and white blood cells (WBC), as well as expression levels of blood urea nitrogen (BUN), creatinine (CRE), glutamic oxaloacetic transaminase (GOT), and glutamic pyruvic transaminase (GPT), of all the experimental mice were analyzed 3 days before treatment (day −3) and right before sacrifice (day 14). For the groups with free CPT and MICFNS ± NIR, the quantities of drug in their tumors and five organs after the 14 day treatment were further analyzed by spectrophotometry at λ = 370 nm.

### 2.14 Statistical Analysis

Statistical analyses were performed using MedCalc software (Version 17.2), and all values were presented as the mean ± standard deviation (SD). Comparisons were analyzed using Student’s *t*-test followed by Dunnett’s post hoc test. Statistical significance was set at *P* < 0.05 throughout the study.

## 3 Results and Discussion

### 3.1 Synthesis and Characterization of MICFNS

**Figure 2:**
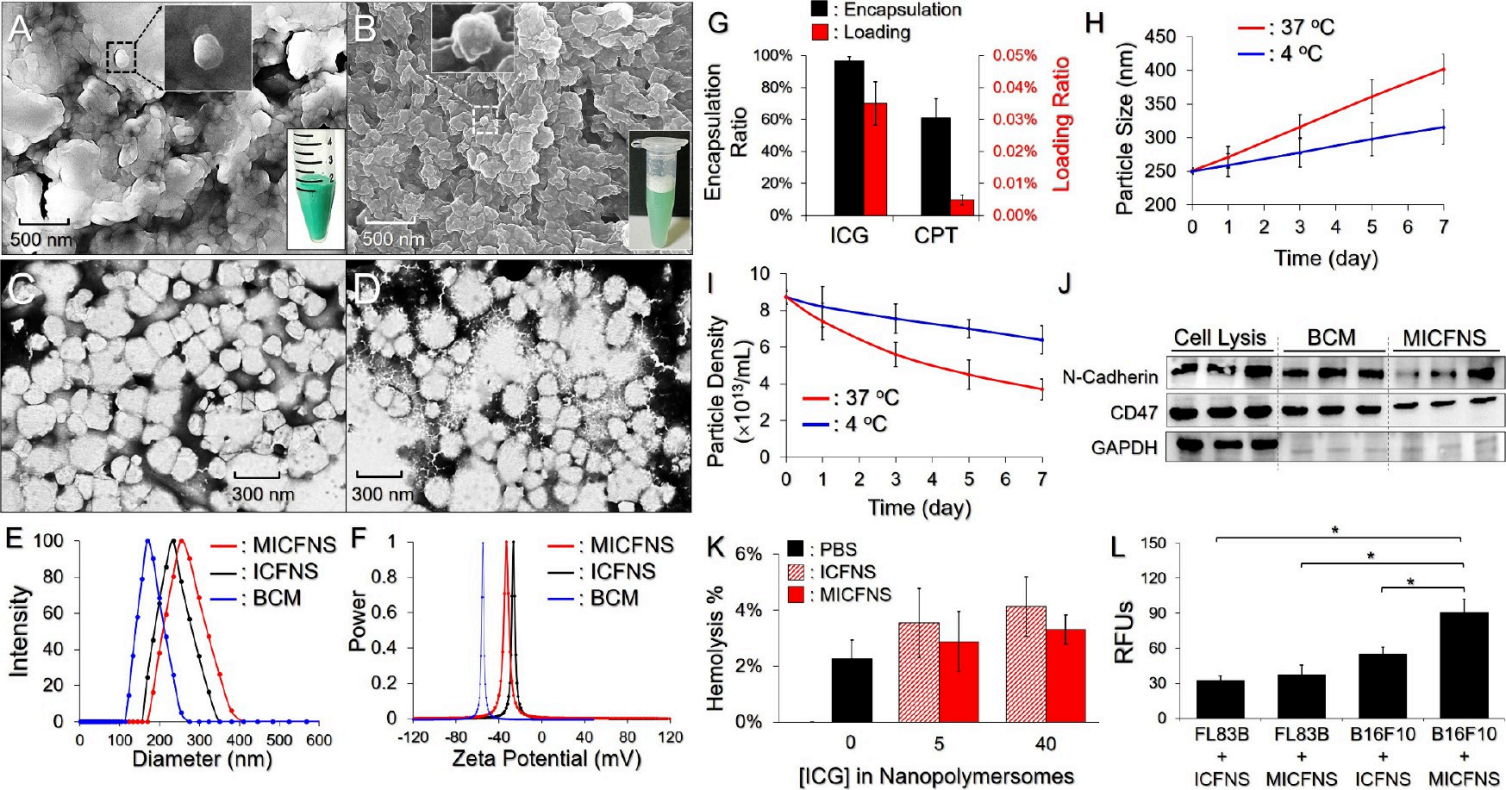
Characterization of the MICFNS. (A, B) SEM images of ICFNS (A) and MICFNS (B) at magnifications of 10,000× and 60,000× (inset). The inset photographs showing green, emulsified solutions exhibit the real appearance of ICFNS (A) and MICFNS (B) samples. (C, D) TEM images of ICFNS (C) and MICFNS (D). (E, F) Representative size distribution (E) and zeta potential (F) of the extruded BCM, ICFNS, and MICFNS measured by DLS. (G) Encapsulation and loading ratios of ICG and CPT in MICFNS. Values are the mean ± SD (*n* = 3). (H, I) Variation of size (H) and density (I) of MICFNS under incubation at 4 or 37 °C for 7 days. Values are the mean ± SD (*n* = 3). (J) Western blot images of N-cadherin, CD47, and GAPDH proteins expressed from the B16F10 cell lysis, BCM, and MICFNS. (K) Hemocompatibility of ICFNS and MICFNS with different doses to murine erythrocytes. Values are the mean ± SD (*n* = 3). (L) Verification of the homology of MICFNS. The intensities of the ICG-derived fluorescence expressed from the FL83B and B16F10 cells were detected by spectrofluorometry performed with 750/838 nm excitation/emission wavelengths and were quantitatively represented by RFUs. Values are the mean ± SD (*n* = 3). *P < 0.05.

Both ICFNS and MICFNS exhibited a green-milky appearance (Figure 2A,B), while MICFNS were formed in granular morphology with a rougher surface compared to ICFNS according to the observations by SEM (Figure 2A,B) and TEM (Figure 2C,D). Based on DLS measurements, the size and zeta potential of the MICFNS were 248.41 ± 8.33 nm with a polydispersity index (PDI) of 0.08–0.15 (Figure 2E) and −32.78 ± 0.29 mV (Figure 2F), respectively, where MICFNS were approximately 18 nm larger than ICFNS (*P* < 0.05) and had a surface charge similar to BCM (*P* = NS), suggesting that the BCM successfully covered the MICFNS surface after the extrusion process. As shown in Figure 2G, the encapsulation efficiencies of ICG and CPT of the MICFNS were 96.64 ± 2.81% and 61.12 ± 11.86%, while their loading ratios were approximately 0.35 and 0.048 wt ‰, respectively.

The stability of MICFNS was evaluated by monitoring their changes in size and density at different temperatures over time. We found that the size of MICFNS increased 26.4% and 60.2% (Figure 2H), while their density decreased 27.2% and 57.6% (Figure 2I), after incubation at 4 or 37 °C for 7 days, indicating that MICFNS were susceptible to increased temperature. We reason that it resulted from reduction of emulsion viscosity and enhanced Brownian motion since heating could increase the particle mobility and thus the rate of collision, leading to breakdown and/or demulsification (e.g., flocculation, Ostwald ripening, and coalescence) of nanopolymersomes as reported previously.^38^

The integrity of the BCM on the MICFNS surface was further identified through Western blot analysis. As shown in Figure 2J, both N-cadherin and CD47 molecules, the two typical transmembrane glycoproteins of the cancer cell membrane,^39,40^ were markedly detected in the BCM as well as MICFNS, demonstrating that MICFNS were certainly covered/camouflaged by the BCM and thereby likely equipped with capabilities of immune escape and cancerous homology according to the functionalities of the two markers reported previously.^41,42^ In addition, GAPDH was barely detected in the MICFNS and BCM compared to that in the cell lysate (Figure 2J) because it is an intracellular/cytosolic marker rather than a membrane protein, resulting in unclear blots in the cell membrane derived samples.

### 3.2 Hemolysis and Homology of MICFNS

Blood compatibility of MICFNS was evaluated by a hemolytic assay using murine erythrocytes. As shown in Figure 2K, the hemolysis due to MICFNS with ≤40 μM of ICG was negligible compared to the result from the group with PBS. In addition, MICFNS can offer a milder hemolytic effect than ICFNS, and we speculate that this was attributable to the decorated BCM since it can provide MICFNS with a cell-like surface. These results demonstrate that MICFNS is equipped with hemocompatibility and may be safely used *in vivo*.

The internalization efficiency of MICFNS for B16F10 cells, which are the source of the BCM, was evaluated by using different cells or drug carriers for comparison. As shown in Figure 2L, the B16F10 cells were found to have 2.4-fold (*P* < 0.05) higher RFUs than FL83B cells after treatment with MICFNS for 4 h. With the same B16F10 cells, on the other hand, the RFUs in the group with MICFNS were 1.6-fold (*P* < 0.05) higher than that those with ICFNS. These results indicate that the MICFNS can be quickly internalized by the homologous B16F10 cells compared to FL83B cells or ICFNS *in vitro*, demonstrating their homology to the parental cells consequently.

### 3.3 Thermal Stability of MICFNS-Encapsulated ICG

**Figure 3:**
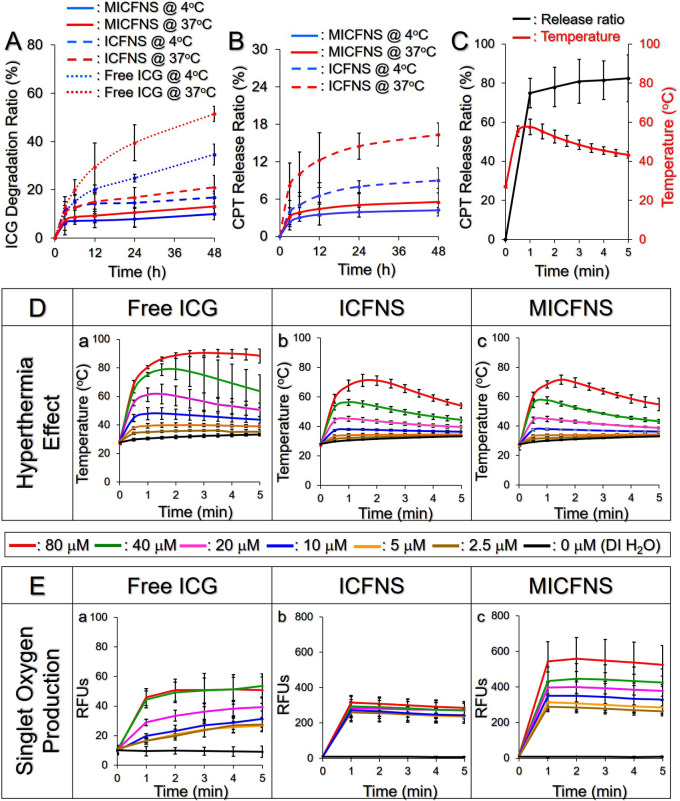
Thermal stability and phototherapeutic functionalities of MICFNS *in vitro*. (A) Degradation efficiencies of ICG dissolved in water or entrapped in ICFNS or MICFNS under incubation at 4 or 37 °C for 48 h. (B) Release kinetics of ICFNS- or MICFNS-encapsulated CPT at 4 or 37 °C within 48 h. (C) CPT release kinetics of MICFNS under NIR exposure for 5 min (black curve). The red curve denotes the variation of temperature in the nanopolymersome system within 5 min NIR irradiation. (D, E) Hyperthermia effect (D) and yields of singlet oxygen (E) produced by free ICG, ICFNS, or MICFNS with various concentrations of ICG under 5 min NIR irradiation. Values in panels A–E are the mean ± SD (*n* = 3).

The thermal stability of ICG entrapped in ICFNS and MICFNS under different temperatures was assessed in this study. As shown in Figure 3A, the degree of ICG degradation in aqueous solution was remarkably higher than that in the ICFNS or MICFNS under the same temperature, while MICFNS exhibited the least ICG degradability at 4 and 37 °C among the three settings. Elevated temperature led to increased degradation of ICG due to its thermal susceptibility.^43,44^ MICFNS were able to save >85% of ICG after incubation at 37 °C for 48 h, and such a maintenance ratio was even higher than the value gained from the free ICG at 4 °C for 48 h. These results demonstrate that MICFNS can offer improved thermal stability of the entrapped ICG compared to naked or ICFNS-entrapped ICG, and we reason that such effectiveness was attributed to the dual protections provided by the nanoencapsulation and surface coating with the BCM.

### 3.4 Release Efficiency of CPT under Various Conditions

Figure 3B shows the CPT release kinetics of ICFNS and MICFNS at various temperatures within 48 h. Both settings displayed a two-phased release profile consisting of a rapid release in the first 3 h followed by a slow sustained release in the rest of the period. The ICFNS were found to have 9.1% and 16.34% of CPT released after incubation at 4 or 37 °C for 48 h, while MICFNS released 4.2% and 5.5%. These outcomes indicate that the coated BCM may reduce the CPT release efficiency because the entangled protein macromolecules of the BCM may form steric hindrances to drug delivery.^45^ Furthermore, the BCM-induced steric hindrances and electrostatic repulsion may reduce the interactions between nanopolymersomes and thus improve the shelf stability of the MICFNS.

The kinetics of CPT release from MICFNS under NIR irradiation was further examined to evaluate the drug release efficiency during phototherapy. As plotted in Figure 3C, the release of CPT was processed in two phases as it was performed without NIR irradiation (Figure 3B), and a cumulative release ratio of 82.5 ± 11.8% was obtained after 5 min. Considering that the temperature of the nanopolymersome system was higher than the melting temperature of PF68 (*T*_m_ ∼ 54 °C) after 60 s NIR irradiation (∼58 °C, Figure 3C), we surmise that at that moment MICFNS were quickly decomposed thereby resulting in bulk release of CPT. These results show that the effect of CPT release from the MICFNS can be greatly facilitated under NIR exposure.

### 3.5 Hyperthermia Effect and Singlet Oxygen Production of MICFNS

Figure 3D illustrates how the free ICG, ICFNS, and MICFNS with different ICG concentrations changed the environmental temperature under NIR irradiation. The results show that all three groups exhibited a biphasic heating profile, in which the two kinds of nanopolymersomes displayed similar outcomes in which only the groups with ≥20 μM of ICG can raise the systemic temperature to >42 °C within the first 90 s of NIR irradiation (Figure 3D(b,c)), indicating that the BCM coating would not affect the hyperthermia effect of the PFC nanopolymersomes. However, the degree of nanocarrier-induced thermal effect was milder than that obtained from the free ICG under the same treatment. We speculate that this result was likely because MICFNS can only partially release ICG, rather than all the ICG molecules freely dissolved in water, to effectuate hyperthermia under NIR irradiation. In addition, PFOB has ∼45.6 kJ/mol enthalpy of evaporation at 1 atm which is higher than that of water (40.65 kJ/mol), indicating that the temperature variation of PFOB emulsions (e.g., MICFNS) is foreseeably lower than that of aqueous solution. Moreover, demulsification and/or decomposition of nanopolymersomes during NIR exposure are endothermic processes that may reduce the enthalpy of the system as reported previously.^46^ Nonetheless, these results clearly demonstrate that MICFNS was certainly able to generate dose-dependent hyperthermia upon NIR irradiation and offer PTT accordingly.

Figure 3E exhibits the singlet oxygen production profiles of the ICG solution (Figure 3E(a)), ICFNS (Figure 3E(b)), and MICFNS (Figure 3E(c)) during 5 min of NIR irradiation. All three formulations were able to quickly produce singlet oxygen within 60 s NIR irradiation, while nanopolymersomes can provide significantly higher yields than free ICG and their productivities can be ranked MICFNS > ICFNS > free ICG. Based on the RFU analysis, the MICFNS were able to produce approximately 10- and 2-fold higher amounts of singlet oxygen compared to free ICG and ICFNS, respectively, when the ICG concentration was ≥40 μM. We speculate that such increased singlet oxygen production in PFC nanopolymersomes was mainly attributed to their hyperoxic character because PFOB can provide exceptional oxygen solubility (53 mmol O_2_/L_PFOB_) compared to water (2.2 mmol O_2_/L_PFOB_).^47^ Furthermore, extrusion during the MICFNS fabrication may bring more air/oxygen into the nanopolymersome system, giving MICFNS a higher singlet oxygen yield compared to that of ICFNS.

### 3.6 *In Vitro* Photochemocytotoxicity of MICFNS

**Figure 4:**
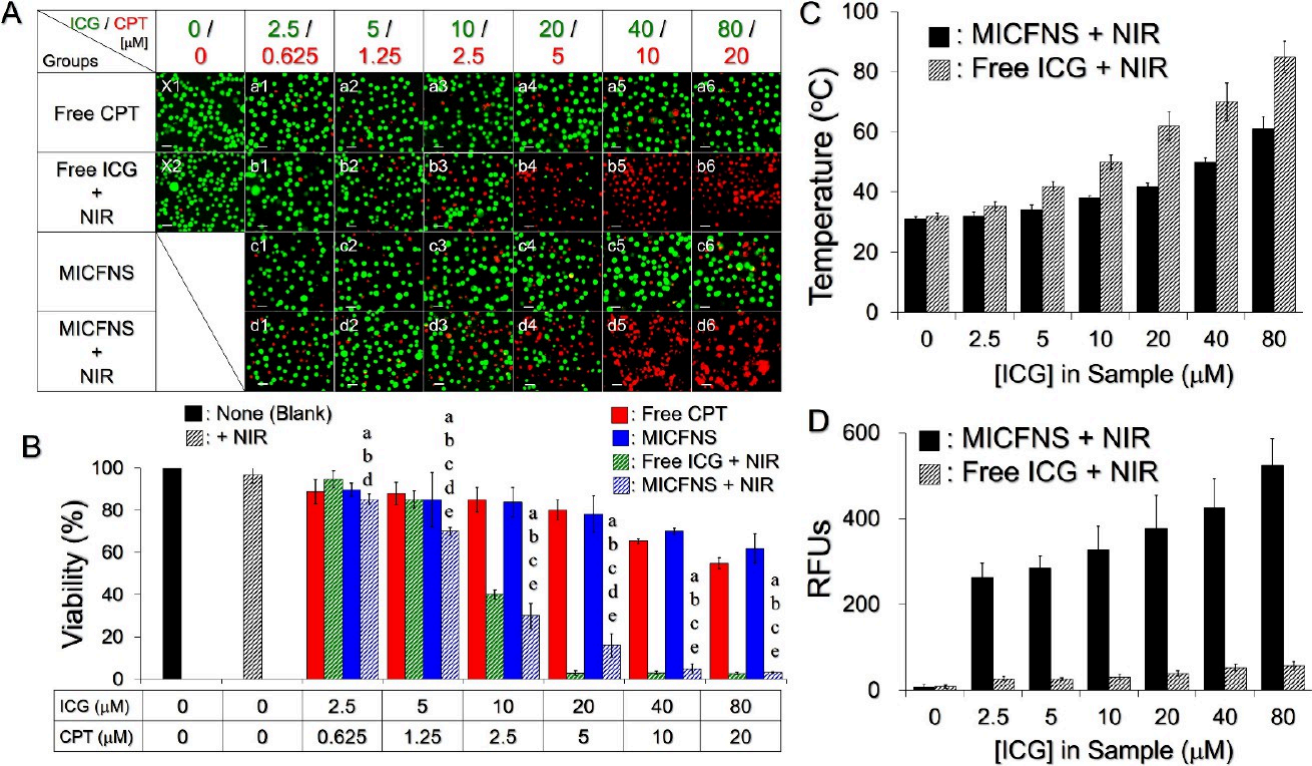
Photochemotherapeutic effects of MICFNS *in vitro*. (A) Fluoromicrographic images of the B16F10 cells 24 h after no treatment (X1; blank control) or treatment with NIR alone (X2), free CPT (a1–a6), free ICG + NIR (b1–b6), MICFNS (c1–c6), or MICFNS + NIR (d1–d6). The doses of ICG and/or CPT provided by different agents were set as [ICG]/[CPT] = 0/0, 2.5/0.625, 5/1.25, 10/2.5, 20/5, 40/10, and 80/20 μM as indicated in the figure. The green and red spots denote live and dead cells stained with calcein-AM and PI, respectively. Scale bar = 100 μm. (B) Quantitative analyses of the viabilities of B16F10 cells 24 h after various treatments. Values are the mean ± SD (*n* = 3). a, b, c, d, and e represent *P* < 0.05 compared to the group treated with none, NIR alone, free CPT, free ICG + NIR, and MICFNS, respectively, under equal ICG and/or CPT dosage settings. (C, D) Hyperthermia effect (C) and yields of singlet oxygen (D) produced from the free ICG or MICFNS with various doses after 5 min NIR irradiation. Values are the mean ± SD (*n* = 3).

Figure 4A shows the microscopic detection of live/dead B16F10 cells 24 h after various treatments. Based on the MTT analysis (Figure 4B), the cells treated with NIR irradiation in the absence of ICG (Figure 4B,X2) exhibited 96.7% viability, demonstrating that the mild increase in temperature caused by NIR irradiation (Figure 3D) was not toxic. On the other hand, dose-dependent cytotoxicity can be observed in all drug-treated groups where we found that (1) MICFNS without NIR enabled a cancericidal effect similar to free CPT under equal dosages, (2) MICFNS + NIR dramatically reduced cell viability to <50% as the dose of ICG/CPT was elevated to ≥10/2.5 μM, and (3) the free ICG + NIR offered the lowest cell viability (<5%) among all settings when its concentration was over 20 μM. We further confirmed that both ICG and MICFNS can generate hyperthermia and singlet oxygen in cells upon NIR exposure as shown in Figure 4C,D, respectively, and those levels were consistent with the data gained from the analyses of MICFNS characteristics (Figure 3D,E). These results illustrate that MICFNS were certainly able to kill cancer cells upon NIR irradiation, and the phototherapy indeed played a critical role in the MICFNS-mediated cancericidal approach. However, the use of free ICG is not a suitable formulation in practice because it is photosensitive/thermally susceptible and easily removed from the circulation^43,44^ that is unfavorable for use *in vivo*.

### 3.7 Tumor Retention and Homology of MICFNS *In Vivo*

**Figure 5:**
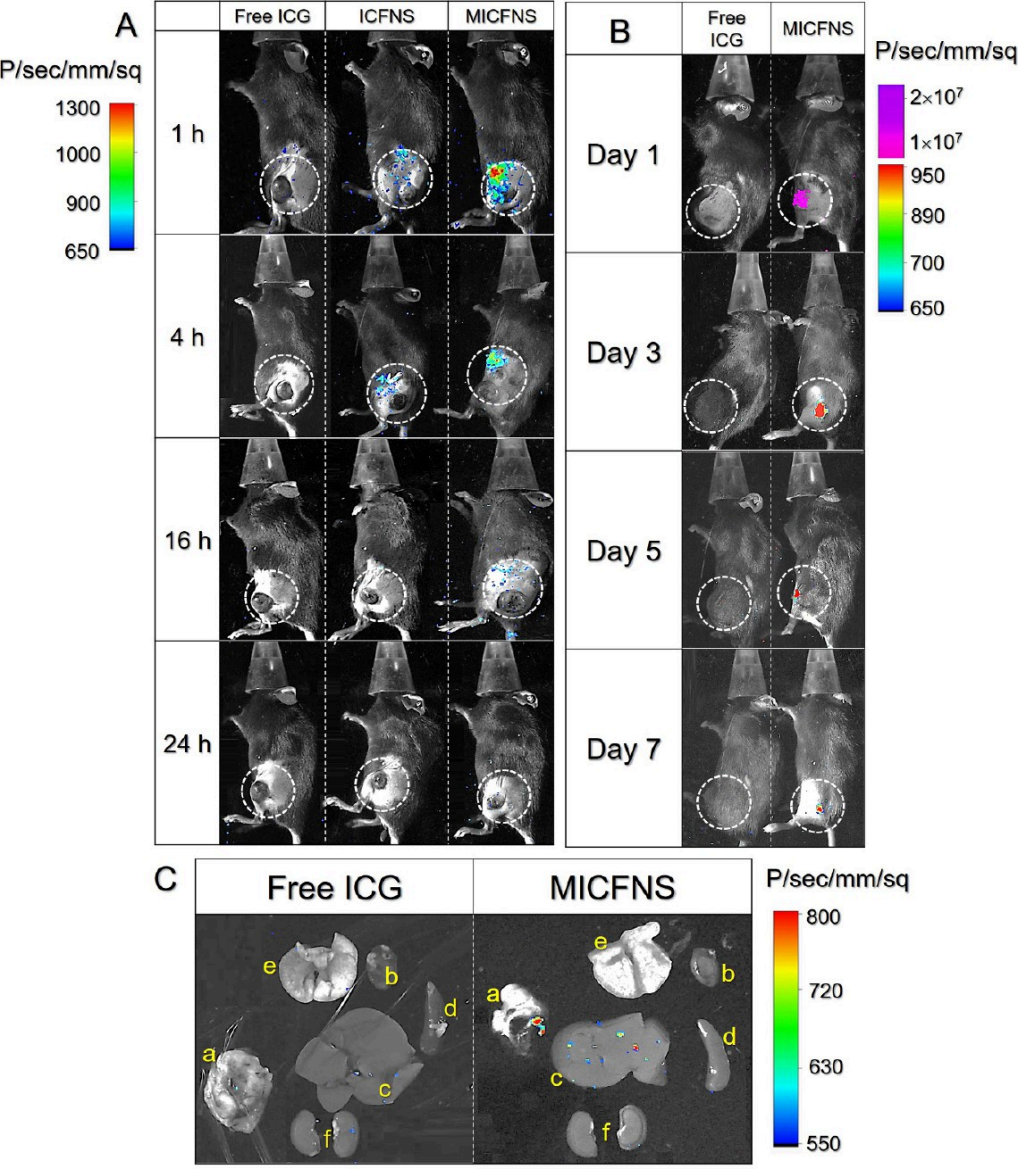
Tumor targeting and retention effectiveness of MICFNS *in vivo*. (A) Time-lapse ICG-derived fluorescent images of the B16F10 tumor-bearing mice after tail intravenous injection of free ICG, ICFNS, or MICFNS within 24 h. (B) Time-lapse ICG-derived fluorescent images of the B16F10 tumor-bearing mice after intratumoral injection of free ICG or MICFNS within 7 days. Dashed circles in parts A and B denote the position of the tumor *in vivo*. (C) ICG-derived fluorescence images of tumors and five organs collected from the mice with intratumoral injection of free ICG or MICFNS after 7 days. a, b, c, d, e, and f represent tumor, heart, liver, spleen, lung, and kidney, respectively.

With confirmed homology of the MICFNS *in vitro* (Figure 2L), the effects of cancer targeting and intratumoral retention of MICFNS *in vivo* were further examined using the ICG-derived fluorescence as the probe. As shown in Figure 5A, no fluorescent signal was found in the group with free ICG beyond the first hour after intravenous injection, while both ICFNS and MICFNS can be detected in tumors, and the latter exhibited a higher quantity of drug accumulation based on the expression level of fluorescence. Such outcomes were reasonably attributed to immune escape and cancer targetability of MICFNS endowed by the BCM surface.^20^ The fluorescent intensities in both types of nanopolymersomes decreased with time, and only MICFNS, but not ICFNS, could be detected after 16 h. Similar results could be obtained in the intratumoral assay that the MICFNS were indeed able to be retained in tumors for a longer time than free ICG, and their fluorescent signal was even detectable after 7 days, while that of free ICG was barely found after 24 h (Figure 5B). Through detecting the fluorescence in organs and tumors excised after sacrifice, our data further manifest that most MICFNS were accumulated in tumors (Figure 5C(a)) rather than in organs (Figure 5C(b–f)) after treatment for 7 days.

### 3.8 Anticancer Effects of MICFNS *In Vivo*

**Figure 6:**
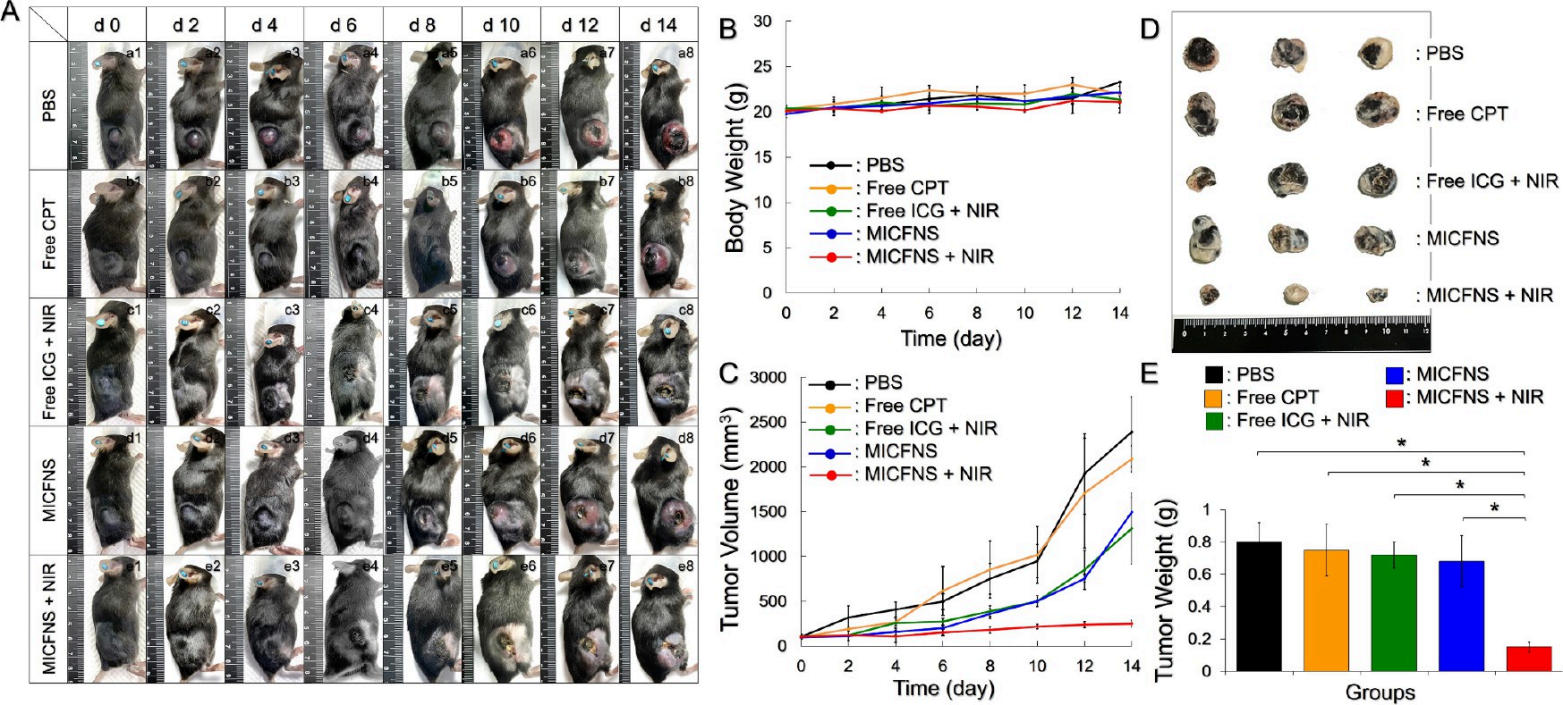
Anticancer effect of MICFNS *in vivo*. (A) Appearance of the B16F10 tumor-bearing mice under various treatments within 14 days. (B, C) Variations of body weight (B) and tumor size (C) of the experimental mice within 14 days. (D) Photograph of all tumors collected after sacrifice. (E) Tumor weight of each group assessed after treatment for 14 days. Values in B, C, and E are the mean ± SD (*n* = 3).

Considering the photochemotoxicity of MICFNS for eradication of cancer cells shown in Figure 4, MICFNS with 40 μM ICG and 10 μM CPT was evaluated in the subsequent animal study. Figure 6A exhibits the conditions of B16F10 tumor-bearing mice with different treatments within 14 days. It can be seen that tumors in the subjects with PBS, free CPT, free ICG + NIR, and MICFNS without light (Figure 6A, rows a–d) dramatically grew, while they were obviously suppressed under treatment of MICFNS + NIR (Figure 6A, row e). No significant body weight change was found in any group within the 14 day treatment (Figure 6B, where *P* = NS). Based on the analysis of tumor size shown in Figure 6C, CPT alone and MICFNS without NIR were not able to inhibit the proliferation of cancer cells, and the tumor sizes dramatically increased by 19.8- and 12.1-fold after 14 days. Free ICG + NIR could effectively destroy tumors, while tumors without NIR exposure successfully grew and resulted in an 11.8-fold increased size of tumor after 14 days. Only MICFNS + NIR could arrest the proliferation of cancer cells, whereby the tumor size was limitedly increased by approximately 2-fold after 14 days. Such exceptional anticancer efficacy performed by MICFNS + NIR can be further verified by visually smaller tumor (Figure 6D) and tumor weight (Figure 6E) compared to all of the other groups. Overall, these outcomes illustrate that MICFNS with 40/10 μM of ICG/CPT in combination with 30 s of NIR irradiation (808 nm; 6 W/cm^2^) was able to arrest the tumor growth *in vivo*.

Based on the analysis of tumor size shown in Figure 6, MICFNS without NIR irradiation were found to have a higher anticancer efficacy than free CPT, and such results could be assumed because MICFNS carrying oxygen by PFOB may alleviate the hypoxia-induced drug resistance. Furthermore, the retention time of MICFNS in tumors was longer than the free agent thus offering a higher antitumor efficacy. In addition, CPT at 10 μM only killed <50% of cancer cells *in vitro* (Figure 4B), implying that this dose of CPT alone is not high enough for use *in vivo*. Nonetheless, CPT is essential in MICFNS-mediated combinational therapy because it can provide sustained anticancer activity to impede tumor recurrence due by the surviving cells after phototherapy, which is an undesired situation that took place in the group with free ICG + NIR. Given that the MICFNS were equipped with both phototherapy and chemotherapy functions, the growth of tumors was successfully arrested by MICFNS + NIR. Furthermore, since MICFNS with 10 μM CPT was demonstrated as an effective dosage for anticancer treatment, MICFNS are not only able to provide antitumor efficacy but also likely to reduce chemotoxicity and/or drug resistance in patients due to the use of less CPT (<IC_50_).

### 3.9 Prognosis of Tumor Development after MICFNS-Mediated Photochemotherapy

**Figure 7:**
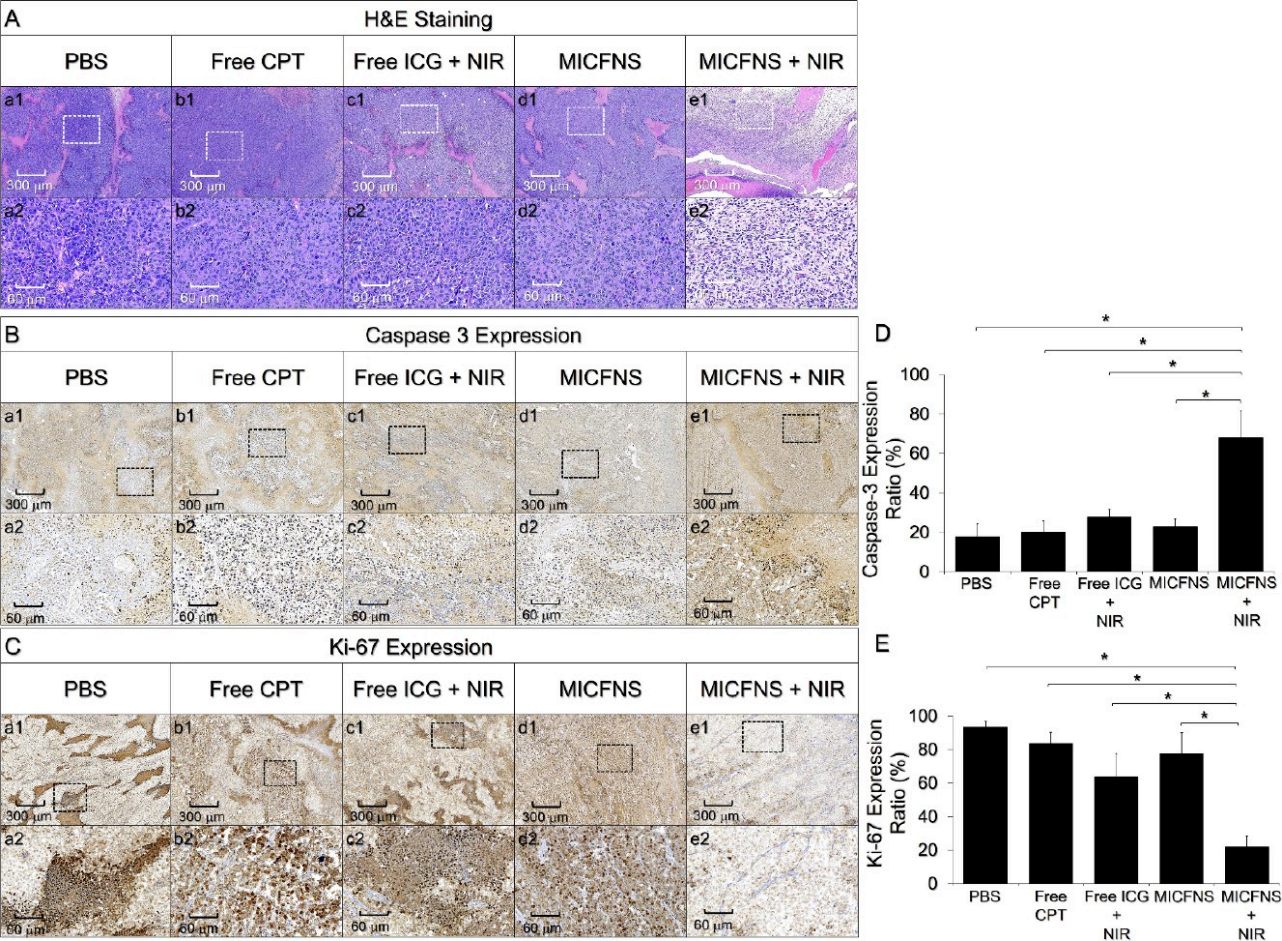
Histological analyses of the tumors collected after sacrifice. (A–C) Representative images of H&E (A), caspase-3 (B), and Ki-67 (C) IHC-staining of tumor tissues for each group. a2–e2 are the magnified images of the areas marked in a1–e1. (D, E) Analyses of expression levels of caspase-3 (D) and Ki-67 (E) in tumor tissues of each group, which were represented by the percentage of the area with marker expression. Values are the mean ± SD (*n* = 3). *P < 0.05.

Tumor tissues of all groups were immediately subjected to H&E staining analysis after sample collection. As illustrated in Figure 7A, all groups except the one with MICFNS + NIR (Figure 7A, column e) showed intensive aggregation of tumor cells (Figure 7A, column a–d), indicating that the tumors in the four groups were formed with high integrity and such results can be reflected by tumor weights presented in Figure 6E.

To predict the tumor progression beyond treatment, the conditions of cancer cells in each group were further assessed through IHC staining for caspase-3 and Ki-67. Caspases are an assembly of signaling molecules involved in cell apoptosis, in which caspase-3 is one of the most studied effectors because it plays a key role in both mitochondrial and receptor-mediated pathways of cell death.^48^ Furthermore, caspase-3 activation is required for induction of cancer cell apoptosis in response to a variety of anticancer drugs including CPT.^49^ On the other hand, Ki-67, also known as MKI67, is a nuclear protein expressed in all proliferating cells but is absent in resting ones (G0 phase),^50^ making it a useful maker for evaluation of cell growth and tumor grading.^51,52^Figure 7B,C displays the expressions of caspase-3 and Ki-67 of all tumor tissues after the 14 day treatment. Based on the analyses of the marker distribution area, the one treated with MICFNS + NIR showed that approximately 70% of the tissues expressed caspase-3 (Figure. 7D) while only ∼20% had Ki-67 signal (Figure 7E), indicating that most of cancer cells in the group underwent apoptosis rather than proliferation after treatment. Tumor development in the other four groups exhibited an opposite tendency, and these data are consistent with the outcomes shown in Figure 6. These results suggest that the incidence of tumor recurrence in the group with MICFNS + NIR would be relatively low; however, more studies are certainly needed to verify the hypothesis.

### 3.10 Systemic Toxicity of MICFNS

**Figure 8:**
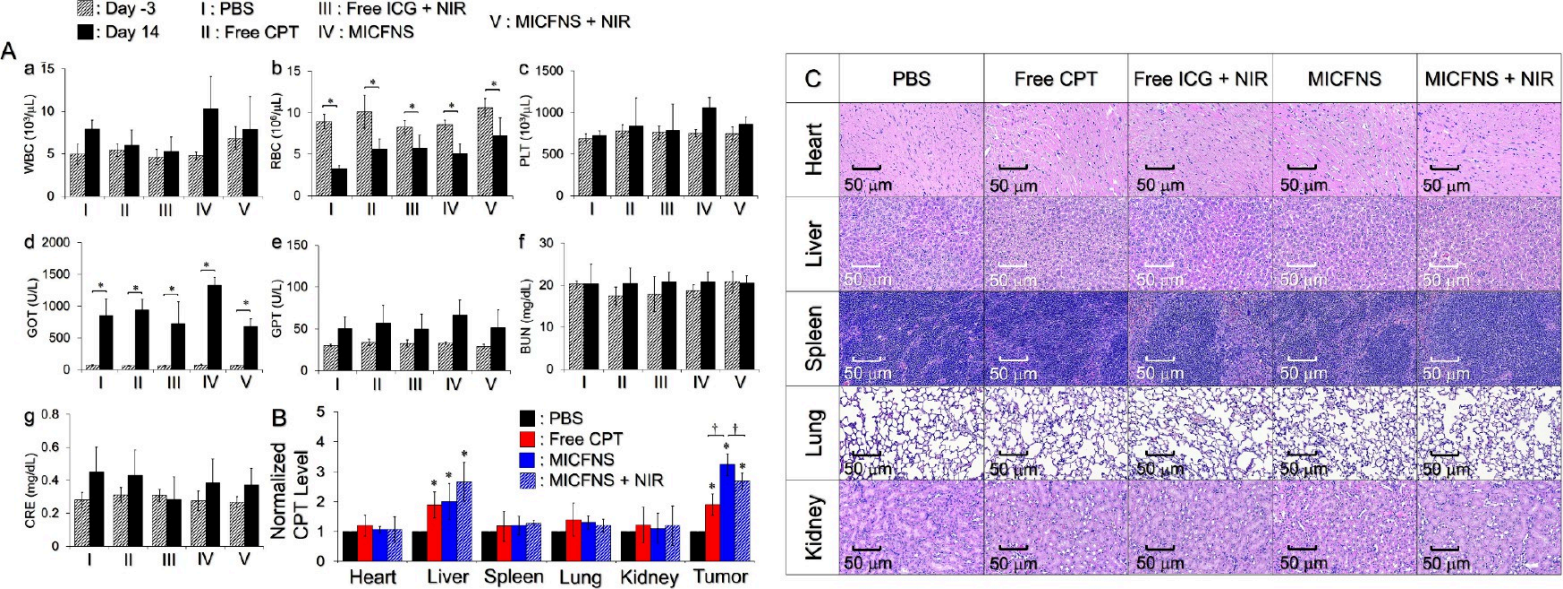
Analyses of systemic toxicity of MICFNS. (A) Numbers of WBC (a), RBC (b), and PLT (c), as well as concentrations of serum markers GOT (d), GPT (e), BUN (f), and CRE (g) of all the experimental mice detected at 3 days before treatment (day −3) and right before sacrifice (day 14). Values are the mean ± SD (*n* = 3). *P < 0.05. (B) Quantitative analysis of the amount of CPT in heart, liver, spleen, lung, kidney, and tumor of the mice after the 14 day treatment. Values are the mean ± SD (*n* = 3). *P < 0.05 compared to the value gained from the group with PBS in the same organ set. ^†^*P* < 0.05. (C) Photomicrographic images of H&E-stained heart, liver, spleen, lung, and kidney tissues excised from the B16F10 tumor-bearing mice with various treatments after sacrifice.

Figure 8A shows the results of serum biochemistry analyses of all of the experimental mice before and after treatments. Our data show that the values of all drug-treated groups were similar to the PBS group under the same time setting (*P* = NS for all), suggesting that the effects of MICFNS + NIR on blood cells (Figure 8A(a–c)), liver (Figure 8A(d,e)), and kidney (Figure 8A(f,g)) of the mice were negligible within the 14 day treatment.

However, it is notable that the RBC numbers (Figure 8A(b)) and GOT levels (Figure 8A(d)) of all groups significantly changed after 14 days. We speculate that this was attributable to the progression of cancer rather than the modality. Indeed, decrease of RBC counts and/or anemia have been identified as a general symptom in various cancers including melanoma.^53^ The inhibitory mechanism is known to be activated because tumor cells can restrict hematopoietic function through bone marrow infiltration and/or induction of iron chelation and production of RBC is seriously inhibited leading to anemia consequently.^54^ Herein the RBC counts decreased in all of the B16F10 tumor-bearing mice, and such circumstance was in line with the clinical syndrome of melanoma. GOT, also called aspartate aminotransferase (AST), is a commonly used serum biomarker for diagnosis of hepatic function, and elevation of GOT often represents potential liver damage.^55^ It has been demonstrated that melanoma is one of the most typical malignancies that would metastasize to liver during the cancer progression,^56,57^ and the increase of GOT indeed complied with this known risk.

For the groups with free CPT and MICFNS ± NIR, the quantities of CPT in the tumors and five organs were concomitantly measured to further evaluate the effects of MICFNS on biological systems. As shown in Figure 8B, except for the liver and tumor where the CPT was directly metabolized and administered,^58^ the three regimens resulted in comparable CPT amounts in the other four organs, and those were all similar to the value from the PBS group (*P* = NS for each). Furthermore, the amount of accumulated CPT in tumors by use of MICFNS was significantly higher than that with free CPT (*P* < 0.05), and such outcomes could be rationalized because the nanopolymersome-encapsulated CPT can be protected from enzymatic attack and/or multiple drug cleanup mechanisms such as transcapillary filtration and/or reticuloendothelial system; furthermore, the retention time of nanopolymersomes in tumors is longer than free molecules (Figure 5B), leading to a greater amount of CPT in tumors. Nonetheless, neither notable lesions nor inflammation was found in the five organs of all drug-treated mice compared with those in the PBS group (Figure 8C). Taken together, these results clearly demonstrate that MICFNS + NIR are highly biocompatible and nontoxic to physiological systems.

## 4 Conclusions

In this study, a type of cancer cell membrane camouflaged PFC nanopolymersomes loaded with ICG and CPT, named MICFNS, was developed for targeting photochemotherapy of cancer. Surface decoration with the cancer cell membrane conferred the capability of immune escape and homology to the nanopolymersomes and was able to further stabilize the entrapped agents. Encapsulated CPT can be quickly released upon NIR irradiation and offers sustained cancericidal effectiveness after phototherapy. MICFNS carrying oxygen by PFOB can promote singlet oxygen production and likely overcome hypoxia-induced drug resistance. MICFNS with ICG and CPT can markedly reduce the effective dosage of CPT and thus alleviate side effects and multidrug resistance which frequently take place in most chemotherapy. Through the animal study using a B16F10 tumor-bearing mouse model, we demonstrated that MICFNS were indeed able to provide homologous cancer targetability and longer drug retention time in tumors compared to ICFNS and free ICG. Moreover, tumor growth can be dramatically inhibited by MICFNS + NIR without notable systemic toxicity within the 14 day treatment. We reasoned that such tumor inhibitory efficacy was produced by phototherapy followed by chemotherapy, a two-stage tumoricidal procedure. Given the aforementioned therapeutic effects together with their safety and bioavailability, the developed MICFNS are anticipated to be a functional nanoplatform for cancer treatment, while the cancer cell membrane on the ICFNS surface could be changed and customized according to the target cancer cells.

## Data Availability

The data used and analyzed during the current study are available from the corresponding author upon reasonable request.
